# The role of age and digital competence on the use of online health and social care services: A cross-sectional population-based survey

**DOI:** 10.1177/20552076221074485

**Published:** 2022-01-28

**Authors:** T Heponiemi, A-M Kaihlanen, A Kouvonen, L Leemann, S Taipale, K Gluschkoff

**Affiliations:** 1 3837 Finnish Institute for Health and Welfare, Helsinki, Finland; 2 Faculty of Social Sciences, 3835 University of Helsinki, Finland; 3 Centre for Public Health, Queen’s University Belfast, UK; 4 Faculty of Humanities and Social Sciences, 4168 University of Jyvaskyla, Finland; 5 Faculty of Social Science, University of Ljubljana, Slovenia

**Keywords:** Digital skills, older people, digital exclusion, online services

## Abstract

**Objective:**

Online health and social care services are getting widespread which increases the risk that less advantaged groups may not be able to access these services resulting in digital exclusion. We examined the combined effects of age and digital competence on the use of online health and social care services.

**Methods:**

We used a large representative population-based sample of 4495 respondents from Finland. Paper-based self-assessment questionnaire with an online response option was mailed to participants. The associations were analyzed using survey weighted logistic regression, exploring potential non-linear effects of age and controlling for potential sex differences.

**Results:**

Higher age, starting from around the age of 60 was associated with a lower likelihood of using online services for receiving test results, renewing prescriptions and scheduling appointments. Good digital competence was able to hinder the age-related decline in online services use, but only up to around the age of 80.

**Conclusions:**

Our results suggest that older adults are at risk of digital exclusion, and not even good digital competence alleviates this risk among the oldest. We suggest that health and social care providers should consider older users’ needs and abilities more thoroughly and offer easy to use online services. More digital support and training possibilities should be provided for older people. It is equally important that face-to-face and telephone services will be continued to be provided for those older people who are not able to use online services even when supported.

## Introduction

Health and social care services are increasingly delivered online. Especially the COVID-19 pandemic has given an unprecedented boost to the digitalization of these services.^1,2^ For example, in the US the use of Telehealth, that is delivery of health-related services through telecommunication and digital technologies, increased from 0.2 per cent in March 2019 to 1.9 per cent in March 2020.^3^ In Finland, the use of remote and phone services regarding psychiatric outpatient visits increased from nine per cent in January to 48 per cent in May 2020, whereas at the same time face-to-face visits declined from 84 to 47 per cent.^4^

Patient portals have become common offering online access to test results, secure messaging, appointment scheduling, and self-care services. Popular online health functionalities include obtaining laboratory or other test results, remote appointments with a doctor or a nurse, health information searching, renewing a prescription, and booking appointments.^5^ Patient portals have been found to evoke positive patient experiences and offer many benefits, such as scheduling flexibility, time savings, and automated reminders that can prevent missing appointments.^6^ The services may also reduce the number of physical health care visits and may therefore mitigate the overall burden on the health care system.^7^ Nevertheless, despite the benefits these services offer, their use poses significant risks because it may increase existing social inequalities and deepen the digital exclusion between those who use the services and those who do not.^8^ Several sociodemographic and socioeconomic factors, such as gender, education and income have been shown to contribute to digital exclusion.^9^

In addition to health care, social care has increasingly moved online. For example, artificial intelligence-assisted moderated online social therapy for youth has shown its viability.^10^ The internet has increasingly been used to deliver interventions,^11^ to treat addictions^12^ and to offer online support.^13^ It has been shown in Finland that the perceptions of potential benefits of online services do not differ much between the clients of health and social care services**.**^14^ However, factors associated with the perceptions differ slightly between these sectors. In health care, education, security concerns and easy access to services were strongly associated with perceptions of the benefits, whereas insufficient amount of online services was highlighted among the clients of social services.

Age is one of the substantial barriers for the use of online services and a key determinant of digital exclusion.^3^ Older adults use the internet much less frequently than younger age groups, both in terms of general use^15,16^ and health-related use.^17^ Older adults are also less likely to use online patient portals than the younger population.^18,19^

Understanding the reasons underlying the age-related digital exclusion is needed for the development of preventive strategies that aim to mitigate exclusion risks. Among those over 65 years of age, older age has been found to be associated with lower likelihood to adopt new information technology.^20^ Moreover, older age has been associated with lower likelihood to own a smartphone and find the internet useful.^19,21^ and may thus have poorer access to online services. Furthermore, they typically have poor digital skills^15^ which may further contribute to their reluctance to use online services.^22^ In addition, cognitive and physical decline may affect the digital use of services especially among the oldest adults.^23^ However, technological modifications, user-centered design and older adults’ needs are often neglected when designing digital appliances and services.^24^

### The present study

Despite the well-established association between older age and exclusion from online services, it remains uncertain at what age the risk of such exclusion begins to increase. Moreover, it is still unclear whether this risk increases at a constant rate or, for example, at an increasing rate with age. Knowledge about how the age-related risk of exclusion from online services develops with age would enrich our understanding of the process of digital exclusion. Previous studies have mainly been limited to small samples, inpatient populations, or to the use of relatively large age categories.^17,18,21,22^ Moreover, with continuous variables it has been assumed that the association between age and online use is linear,^16^ but the nature of this association should be studied more precisely.

It would be important to identify more thoroughly the interaction between age and digital competence for the usage of online services. It is not known whether age is an independent risk factor for exclusion from online services when the effects of digital competence are accounted for. Moreover, it is unclear to what extent having good digital competence may buffer against the age-related risk of digital exclusion. That is, whether age is a risk factor for digital exclusion only if the digital competence is low. Finding answers to above mentioned questions would help to develop more effective interventions addressing lower use of online health and social care services among older adults.

In the light of the above-mentioned knowledge gaps, we aim to examine the combined effects of age and digital competence on the use of online health and social care services in a large representative population-based sample. More specifically, first we aim to examine whether age is related to online service use and at which age this possible effect might start and how does the association evolve when the age increases. Second, we examine whether digital competence might have the capacity to buffer against the possible negative effects of age on online service use. We focus on the most commonly used health and social care services, that is, online use of receiving test results, renewing of a medical prescription, scheduling an appointment, and having an appointment.

## Methods

### Sample

We drew a sample from the Population Register of Finland including 10,000 people representative of the adult population (aged 20 years and above) living in Finland. To guarantee a sufficient number of older respondents, we oversampled 75-year-olds or older. More detailed information on how the data was gathered can be found elsewhere.^5^ The paper questionnaire used for data collection included an online response option. The questionnaire and invitation to participate were mailed in 2017. A maximum of three reminders were sent out for those who had not responded. Ethical approval for the study was received from the Research Ethics Committee of the Finnish Institute for Health and Welfare (THL/637/6.02.01/2017).

Altogether 4495 people responded (response rate 45%). Of the responses, 1298 were answered online (28.9%) and others by paper. Compared to the eligible population, the sample was older, more likely female, and more educated.^5^ Inverse Probability Weighting (IPW) was used to reduce possible non-response bias.^25^

### Measurements

#### The use of online health and social welfare services

The respondents were asked if, during the past year, they had used different health and social care services traditionally (paper, visit or phone call) or online (via mobile devices or computer). The response options were 1) no, 2) yes, traditionally, and 3) yes, online. We identified the most popular types of health and social care services that had been used (either traditionally or online). These were: Receiving laboratory or other test results; Requesting a renewal of a prescription; Scheduling a health or social care appointment and Having an appointment with a health or social care professional. A service-specific binary variable for online use (1 = yes, 0 = no) was coded as follows: respondents who had selected the “yes, online” response options were classified as online users for that service; respondents who had selected the “yes, traditionally” response options were classified as non-online (i.e. traditional) users for that specific service. Respondents who had not used the services (i.e. had selected the response option “no”) were not considered in the study. It was possible also to select both traditional and online use and these respondents who reported both ways of use of a specific service were excluded given their low amount (1% to 5% of the users, depending on the service).

#### Digital competence

Digital competence was measured with one item, “*What is your assessment of your ability to use the internet - online services (on computer or mobile devices)*?” with response options (1) I do not use them; (2) novice (I use them with assistance); (3) I use the basic services independently; (4) I use many online applications effortlessly; and (5) expert (I can teach others). These original five response categories were collapsed into three categories: poor (options 1 and 2), average (option 3) and good competence (options 4 and 5).

#### Demographic characteristics

Demographic characteristics included age (divided by 10 to improve interpretation of the results) and sex (1 = male, 2 = female).

### Statistical analysis

After identifying the most commonly used health and social care services, subsamples consisting of service users were formed from the whole sample, one for each service (*received laboratory or other test results,* n = 2545; *requested a renewal of a medical prescription,* n = 2331; *scheduled a health or social care appointment*, n = 2284; *had an appointment with a health or social care professional*, n = 2140 users). Respondents who reported both traditional and online use of a specific service were excluded (1% to 5% of the users, depending on the service). Furthermore, those with incomplete data on age, sex, or digital competence were excluded, resulting in analytic subsample sizes ranging from n = 2003 to n = 2403 (see Table 1).

**Table 1.** Descriptive statistics for the subsamples.

	Received test results	Renewed a prescription	Scheduled an appointment	Had an appointment
Age (M, (SD))	53.22 (17.73)	55.99 (17.28)	50.67 (17.48)	49.91 (17.93)
Sex				
male	42%	42%	42%	41%
female	58%	58%	58%	59%
Digital competence				
poor	21%	25%	17%	18%
average	24%	25%	23%	23%
good	55%	49%	60%	59%
Online use				
yes	47%	43%	54%	2%
no	53%	57%	46%	98%
Sample size	2403	2190	2003	2271

*Note.* Received test results = Received laboratory or other test results; Renewed a prescription = Requested a renewal of prescription; Scheduled an appointment = Scheduled a health or social care appointment; Had an appointment = Had an appointment with a health or social care professional.

The associations of age and digital competence with the online use of each specific health and social care service were analyzed using survey weighted logistic regression, controlling for potential sex differences. First, only the effects of age on online service use were examined. In addition to a linear term for age, quadratic, or cubic terms were includedNext, the main effects of age and digital competence on online service use were examined. In the final step, the interaction between age and digital competence was tested. Only interactions with linear term for age were considered to avoid fitting a too complex model. The analyses were conducted in R version 3.6.1.^26^

## Results

### Descriptive statistics

Descriptive statistics for the subsamples are shown in Table 1. The mean age ranged from 50 to 56 years and women were slightly overrepresented in the subsamples (from 58% to 59%). The majority of respondents rated their digital competence as good (49% to 60%). Higher age was associated with poorer digital competence (see Supplementary Figure 1). Whereas having an online (vs. a traditional face-to-face) appointment was very rare, using other online services such as renewing a prescription was relatively common.

### The associations of age and digital competence with the use of online services

**Figure 1.**
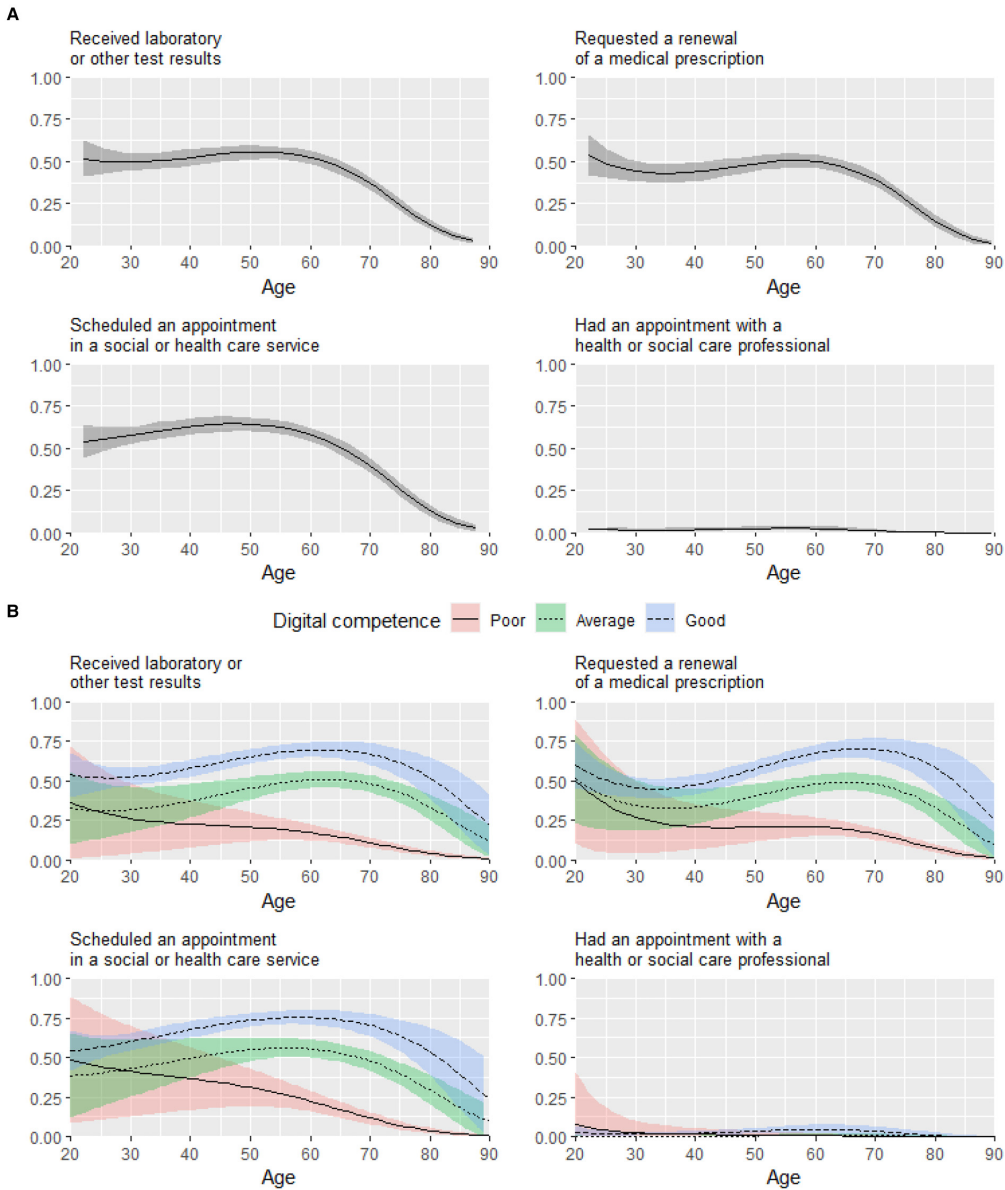
The association between age and the online use of health and social care services in general (A) and by levels of digital competence (B). The y axis shows the probability of online (vs traditional) service use.

The results of the regression analysis are presented in Table 2 and Figure 1. It should be noted that in a nonlinear model such as logistic regression, the effect of a predictor varies across its values, even in the absence of interaction terms. In addition to predicted probabilities in Figure 1, we therefore also present the results visually as average marginal effects in Supplementary Figure 2.

**Table 2.** The results of regression analysis.

Step	Variable	Received test results	Renewed a prescription	Scheduled an appointment	Had an appointment
		OR	OR	OR	OR
**Step 1**					
	Age	0.14*	0.03***	0.48	0.01
	Age^2	1.69***	2.25***	1.35	3.07
	Age^3	0.96***	0.94***	0.97**	0.92
Pseudo *R^2^*		7%	5.6%	7%	3%
**Step 2**					
	Age	0.15*	0.02***	0.45	1.01
	Age^2	1.66**	2.38***	1.38	3.02
	Age^3	0.96***	0.94***	0.97*	0.92
	Digital competence				
	poor	-	-	-	-
	average	5.86***	4.23***	4.86***	1.16
	good	12.61***	8.82***	10.91***	6.48
Pseudo *R^2^*		14.5%	12.4%	13.6%	6.9%
**Step 3**					
	Age	0.13*	0.02***	0.33	0.01
	Age^2	1.55*	2.23***	1.29	2.88
	Age^3	0.97**	0.95***	0.98	0.93
	Digital competence				
	poor	-	-	-	-
	average	0.35	0.59	0.27	0.00
	good	0.89	0.66	0.44	0.07
	Age* poor digital competence	-	-	-	-
	Age* average digital competence	1.55**	1.34	1.59**	2.75*
	Age* good digital competence	1.52*	1.51**	1.70**	2.11
Pseudo *R^2^*		14.8%	12.6%	14.0%	7.2%

*p<0.05 **p<0.01 ***p<0.001

Age was associated with all types of online service use except with having an online appointment. The association between age and the use of online services was nonlinear with a greater age-related decrease in the rate of online use among older adults from around 60 years of age (see Figure 1, panel A). For example, the predicted probability of online service use in terms of receiving laboratory or other test results was relatively high for 40-year-old and 60-year-old individuals (52.42%, 95% CI 47.43–57.41 and 52.34%, 95% CI 48.44–56.23, respectively), but much lower for 80-year-old individuals (12.52%, 95% CI 9.70–15.35). The associations of age remained statistically significant after controlling for digital competence. Overall, the association between good (vs. poor) digital competence and online service use was strong: OR = 12.61 (95% CI 8.52–18.64) for receiving laboratory or other test results, OR = 8.82 (95% CI 6.15–12.64) for renewing a prescription, OR = 10.91 (95% CI 7.24–16.44) for scheduling an appointment, and OR = 6.48 (95% CI 0.93–45.12, not significant) for having an appointment.

There was a statistically significant interaction between age and digital competence on the online use of all types of services, except for having an appointment. Compared to poor level of competence, better digital competence was associated with a higher likelihood of online service use from midlife onwards (from around 40 years of age). However, among older adults (from around 70 to 80 years of age), the association between good digital competence and online service use attenuated with most of the services.

## Discussion

The present study examined the combined effects of age and digital competence on the use of online health and social care services. We found that increasing age was associated with lower likelihood of using online services for receiving test results, renewing medical prescription and scheduling an appointment, but mainly only among older adults starting approximately from the age of 60. However, good (or at least average) digital competence was able to hinder this age-related decline of online service use until around the age of 80. Thus, even good digital competence did not help to keep the use levels high among the oldest respondents.

Our results suggest that older adults are in danger of digital exclusion. This is congruent with previous findings showing an age-related decline in using online services and the internet in general^15–19^ The above mentioned previous studies have mainly used age groupings, which makes it hard to point out when the actual decline in use starts and how it progresses. Our findings show that the association between age and online service use is nonlinear. Age is unrelated to online use before the age of 60, but after that older adults are less likely to use online services as they get older. A previous study points to the same direction showing that the interest in using technology for health, smartphone ownership and eHealth literacy levels were lower for those over 75 years of age compared to those between 50 and 64.^27^

In the present study, age was associated with online service use of receiving test results, renewing prescriptions and making appointments, but not with having online appointments. This finding is of importance because digital exclusion is often considered to concern older adults and there is a risk that older people drop out from essential health and social care services when these services are increasingly delivered online. Our finding that age was unrelated to having online appointment gives some reassurance that older adults might still get their appointments with health and social care professionals even though these appointments increasingly take place online. However, when interpreting this finding it is important to note that only small share of respondents actually used online appointments, thus this finding can only be considered as preliminary and needs to be confirmed in future studies.

Digital competence was able to mitigate the age-related decrease in the use of online health and social care services until the very old age. This finding supports the suggestion that digital competence is crucial for ensuring the adoption of personal health records which may include, for example, patient-reported outcome data, lab results, and data from devices.^28^ However, it is worrisome that according to our results higher age is associated with poorer digital competence. Similarly, in the European Union 80 per cent of the interviewed 44–54 year olds rated themselves as digitally skilled in their daily life compared to 44 per cent of those 65 years and older.^15^ For older adults, the use of online services requires learning a wide range of new skills, starting with the use of touch screens on smart devices, thus overcoming the digital divide requires investing in developing these skills. Various interventions, such as educating young people to mentor older individuals^29^ or games that develop motoric and cognitive skills required to use smart devices,^30^ for example, have been found to be promising. We found, however, that among the oldest respondents, good digital competence was not anymore able to mitigate the age-related decline in the use of online services. A previous study from the US showed that 75–79 year-olds were less likely than 65–69 year-olds to have the required digital tools, skills, and experience to take advantage of new web- and mobile-based modalities for obtaining health-related information and advice.^31^ Our study adds to this by showing that even those oldest old who have good digital competence do not use online services.

There are many possible reasons why among the oldest respondents even good digital competence does not guarantee a shift from traditional service use to online service use. Cognition, motivation, physical ability and perceptual problems have been identified as main barriers for use among older people.^32^ Moreover, privacy and security concerns, lack of access to technology and financial issues may have an effect.^23^ In a previous study, older people were found to be motivated to use information technology but their preference for human contact, fear of consequences and usability issues prevented successful engagement.^33^ Another previous study similarly suggested that older adults’ preferred method of communication involves physical face-to-face interactions and traditional codes of etiquette.^34^

According to our results it seems that until about the age of 60 digital competence is unrelated to online service use. Thus, there must some other reasons than calendar age for non-use among younger generations. Maybe other barriers of online use such as lack of trust, availability of services, negative attitudes and quality issues have a role here.^5^ It might also be possible that practically almost all those under 60 years of age in Finland would already have sufficient competence for using online services, thus even though self-rated competence would be low it would not affect the use. Moreover, a substantial part of health care services among the working-age population in Finland are delivered by occupational health care which may be more often provided online.

### Strengths and limitations

We were able to use a large sample representative of the Finnish adult population. Moreover, we were able to identify and examine various widely used online services. A further strength is that we explored both linear and non-linear associations of age.

However, there are some limitations that need to be considered. We used self-reported cross-sectional data. Thus, the problems associated with common method variance and inflation of the strengths of associations might be an issue here. Our digital competence measure included only one item, thus it was rather crude measure of digital competence. If competence would have been assessed with a measure which would include different dimensions of digital competence, we could have given more detailed information of which aspects of digital competence are of importance. This would be important to study in the future.

Due to cross-sectional data we could not detect causal relationships. We controlled for sex but, however, there is always a possibility of residual confounding. For example, factors such as personal characteristics, cognition, language proficiency, motivation and physical ability may have a big effect here and affect especially older adults’ online use. Therefore, future studies should examine the effects of these factors in this context and whether, for example, the effects of age and competence for online use is partly explained by these factors. The cohort effect may be one driving force related to our results. Younger generations are more familiar with using digital appliances and use them more fluently, whereas older generations are less likely to adopt new information technology^20^ and own digital appliances.^19,21^ However, according to our results, after reaching very old age not even good digital competence helps in using online services. Thus, it seems that even though the cohort effect is important, the actual age matters highly especially in the old age and factors related to old age such as cognitive and physical decline may hinder the use or make it very difficult.^23^

Our sample included more older people, women, and highly educated people than the eligible population.^5^ Therefore, we used IPW which has previously been shown effective in reducing possible non-response bias in Finnish population studies.^25^ Even though we oversampled those 75-year-olds or older, our sample may be biased in a way that especially those oldest persons with dementia or otherwise bad health might have not answered, for example, because low functionality. Finland is among the forerunners of digitalization of health and social care services, thus generalizing our findings to other countries should be done cautiously. However, differences between Finland and other Nordic countries and many Western Europe countries are not that crucial that some kind of comparison would not be possible.

### Implications

The number of new online health, social and other public services is increasing rapidly. The use of these services requires self-management, self-service and a more active role from the clients. Especially the oldest old are at risk of digital exclusion. Therefore, some means should be implemented to increase the online service use among older people. One way to increase the use among older people is offering them support in using online services. For example guidance and support from family members may increase the use of online services among older people.^35^ However, given that many older adults may have difficulties finding somebody to help them, it would be important that public sector would offer help and support in using online services.

Positive experiences about online services and getting benefits from those services might help to increase the motivation and lower the threshold to use online services. Hedonic motivation reflecting joy to use has been shown an important predictor of technology adoption.^36^ The clients have been found to appreciate the e-booking system mainly because of the benefits they derive from it, namely, scheduling flexibility, time savings, and automated reminders that prevented forgetting appointments.^6^

There are also barriers of use related to the technology itself and most of the appliances and online services are not designed to take into account the unique needs of older adults who may have many physical and cognitive limitations. This can have a strong impact on the use of online services among older adults. Friemel (2014) has highlighted that technological improvements are vital to address the age-related barriers affecting older adults’ technology use. To promote positive user experiences and support clients it is important to focus on better technical solutions, usability, and user's needs when designing solutions.^37^ Better usability of the systems might increase older people's confidence in managing their health matters online which is likely to increase the use.^38^

Digital competence seems to be crucial for the use of online health and social care services when people pass middle age. Thus, it would be important to provide training and education for older people.^39^ Competence and skills among older people can be improved, for example, by ICT training including practicing in pairs, possibility to practice with the device, the possibility to influence what to learn and the availability of material that enables communication and learning.^29^ Still, further information is needed on effective ways how to strengthen the required digital competence among older people in order to promote their equal access to, and opportunities to benefit from, various online services.

However, our findings show that traditional services, such as face-to face services, should not be completely replaced by online services. This is especially important for the oldest old. In our study not even good digital competence was able to buffer against low use of online services among the oldest participants Therefore, it is of utmost importance that health and social care organizations continue to offer their services also by other means than online, so that they can give special attention and face to face service to groups that most need it.

### Conclusions

We examined the most commonly used health and social care services and showed how the proportion of individuals who use these services online varies with age. In addition, we highlighted how age-related online service use depends on the level of digital competence. Our results suggest that older adults are at risk of digital exclusion, and not even good digital competence helps to alleviate this risk among the oldest More specifically, after the age of 60, higher age was associated with lower use of online services such as receiving test results, renewing prescriptions and making appointments. However, there was no relationship between age and having online appointments. Digital competence was associated with higher online use among midlife and those 60 to 80 years old, but not among the oldest participants. Thus, good digital competence may promote using health and social care services online among older adults but does not alleviate the risk of exclusion from the services for the oldest if the services are provided exclusively online.

To mitigate the age-related digital exclusion, we suggest that health and social care service providers should focus on technically stabile, easy to use online services which help to motivate the use particularly among older people. High age users’ needs and abilities should be considered before implementing online services. Moreover, digital support and training possibilities should be provided for older people, not only by the third sector organizations, but also by the public sector. However, we highlight that traditional such as face-to-face and telephone services will be needed to guarantee accessibility of essential services also to those older people who are not able to use online services.

## Supplemental Material

sj-docx-1-dhj-10.1177_20552076221074485 - Supplemental material for The role of age and digital competence on the use of online health and social care services: A cross-sectional population-based survey Click here for additional data file. Supplemental material, sj-docx-1-dhj-10.1177_20552076221074485 for The role of age and digital competence on the use of online health and social care services: A cross-sectional population-based survey by T Heponiemi, A-M Kaihlanen, A Kouvonen, L Leemann, S Taipale and K Gluschkoff in Digital Health

sj-docx-2-dhj-10.1177_20552076221074485 - Supplemental material for The role of age and digital competence on the use of online health and social care services: A cross-sectional population-based survey Click here for additional data file. Supplemental material, sj-docx-2-dhj-10.1177_20552076221074485 for The role of age and digital competence on the use of online health and social care services: A cross-sectional population-based survey by T Heponiemi, A-M Kaihlanen, A Kouvonen, L Leemann, S Taipale and K Gluschkoff in Digital Health
