# Supplementary material for: The role of age and digital competence on the use of online health and social care services: A cross-sectional population-based survey
Source: Digit Health. 2022 Jan 28;8:20552076221074485. doi: 10.1177/20552076221074485 (PMC8801649; doi:10.1177/20552076221074485)
Supplement: sj-docx-1-dhj-10.1177_20552076221074485 - Supplemental material for The role of age and digital competence on the use of online health and social care services: A cross-sectional population-based survey [file sj-docx-1-dhj-10.1177_20552076221074485.docx]

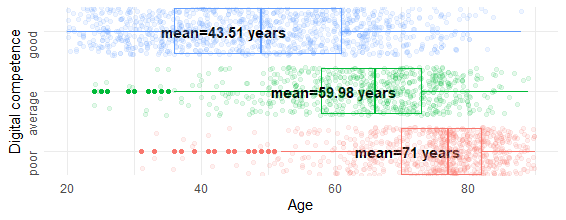


**Supplementary Figure 1.** The association between age and digital competence among respondents who had received test results: boxplots and mean age by the level of digital competence. According to one-way ANOVA, there was a significant difference in mean age [F(2,2400)=949.3, p < 0.001] between the digital competence levels.
