# Supplementary material for: The role of age and digital competence on the use of online health and social care services: A cross-sectional population-based survey
Source: Digit Health. 2022 Jan 28;8:20552076221074485. doi: 10.1177/20552076221074485 (PMC8801649; doi:10.1177/20552076221074485)
Supplement: sj-docx-2-dhj-10.1177_20552076221074485 - Supplemental material for The role of age and digital competence on the use of online health and social care services: A cross-sectional population-based survey [file sj-docx-2-dhj-10.1177_20552076221074485.docx]

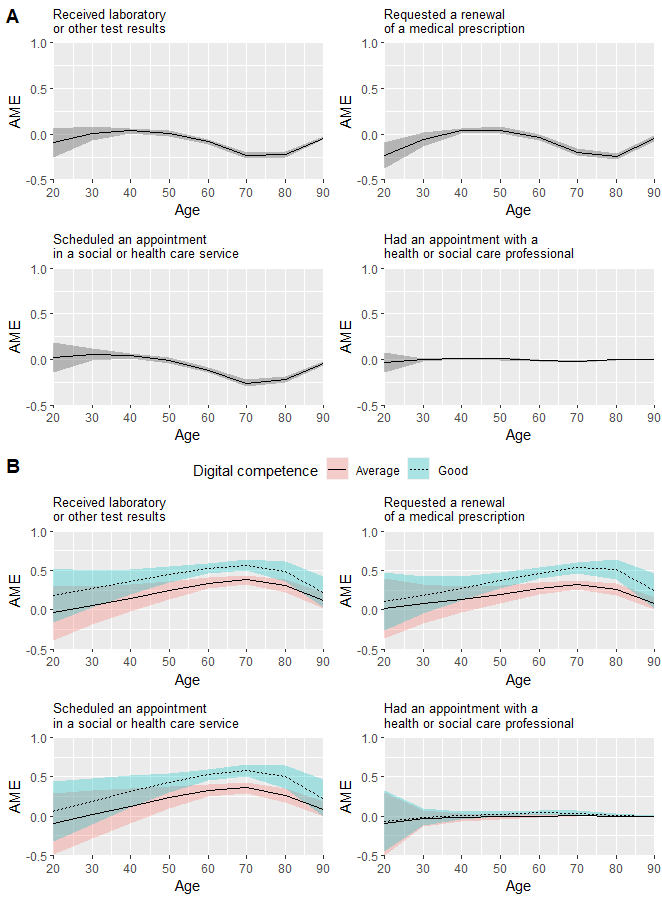


**Supplementary Figure 2**. Average marginal effects (AME) of age (A) and digital competence (B) on the probability of online (vs traditional) use of health and social care services.
